# Pre-radiotherapy plasma carotenoids and markers of oxidative stress are associated with survival in head and neck squamous cell carcinoma patients: a prospective study

**DOI:** 10.1186/1471-2407-9-458

**Published:** 2009-12-21

**Authors:** Amrit K Sakhi, Kjell M Russnes, Magne Thoresen, Nasser E Bastani, Anette Karlsen, Sigbjørn Smeland, Rune Blomhoff

**Affiliations:** 1 Department of Nutrition, Institute of Basic Medical Sciences, University of Oslo, 0316 Oslo, Norway; 2 Division of Cancer Medicine and Radiotherapy, Norwegian Radium Hospital, Oslo University Hospital, Montebello, NO-0310 Oslo, Norway; 3 Department of Biostatistics, Institute of Basic Medical Sciences, University of Oslo, 0317 Oslo, Norway

## Abstract

**Background:**

The purpose of this study was to compare plasma levels of antioxidants and oxidative stress biomarkers in head and neck squamous cell carcinoma (HNSCC) patients with healthy controls. Furthermore, the effect of radiotherapy on these biomarkers and their association with survival in HNSCC patients were investigated.

**Methods:**

Seventy-eight HNSCC patients and 100 healthy controls were included in this study. Follow-up samples at the end of radiotherapy were obtained in 60 patients. Fifteen antioxidant biomarkers (6 carotenoids, 4 tocopherols, ascorbic acid, total antioxidant capacity, glutathione redox potential, total glutathione and total cysteine) and four oxidative stress biomarkers (total hydroperoxides, γ-glutamyl transpeptidase, 8-isoprostagladin F_2α _and ratio of oxidized/total ascorbic acid) were measured in plasma samples. Analysis of Covariance was used to compare biomarkers between patients and healthy controls. Kaplan-Meier plots and Cox' proportional hazards models were used to study survival among patients.

**Results:**

Dietary antioxidants (carotenoids, tocopherols and ascorbic acid), ferric reducing antioxidant power (FRAP) and modified FRAP were lower in HNSCC patients compared to controls and dietary antioxidants decreased during radiotherapy. Total hydroperoxides (d-ROMs), a marker for oxidative stress, were higher in HNSCC patients compared to controls and increased during radiotherapy. Among the biomarkers analyzed, high levels of plasma carotenoids before radiotherapy are associated with a prolonged progression-free survival (hazard rate ratio: 0.42, 95% CI: 0.20-0.91, p = 0.03). Additionally, high relative increase in plasma levels of d-ROMs (hazard rate ratio: 0.31, 95% CI: 0.13-0.76, p = 0.01) and high relative decrease in FRAP (hazard rate ratio: 0.42, 95% CI: 0.17-0.998, p = 0.05) during radiotherapy are also positively associated with survival.

**Conclusions:**

Biomarkers of antioxidants and oxidative stress are unfavourable in HNSCC patients compared to healthy controls, and radiotherapy affects many of these biomarkers. Increasing levels of antioxidant biomarkers before radiotherapy and increasing oxidative stress during radiotherapy may improve survival indicating that different factors/mechanisms may be important for survival before and during radiotherapy in HNSCC patients. Thus, the therapeutic potential of optimizing antioxidant status and oxidative stress should be explored further in these patients.

## Background

Mammalian cells are constantly exposed to oxidative stress and rely on a comprehensive array of antioxidant defence comprising of both enzymatic and non-enzymatic molecules [1]. Among non-enzymatic molecules, both endogenous- (e.g. thiol-containing compounds like glutathione) and dietary- (e.g., phytochemicals like carotenoids, vitamin E, vitamin C and polyphenols) low molecular weight antioxidants have shown to effectively neutralize reactive oxygen species (ROS) and reactive nitrogen species (RNS). Dietary antioxidants that work complementary to endogenous antioxidants in removing free radicals directly [2-4], may also up regulate different genes involved in the endogenous antioxidant defence [5].

It is hypothesized that oxidative stress is involved in the pathogenesis of different types of cancer [6-8]. Head and neck squamous cell carcinoma (HNSCC) has a strong link to oxidative stress since tobacco and alcohol are clearly defined as etiologic factors for these malignancies [9,10]. Furthermore, several studies have shown that the risk of developing HNSCC is linked to low intake of fruits and vegetables, certain genotypes of the enzymes glutathione S-transferases (GSTs) and human papillomavirus (HPV) infection [10-13]. All of these factors are known to increase ROS and RNS production, and to induce cellular oxidative damage.

Radiotherapy is a cornerstone in the treatment of HNSCC. Ionic irradiation exposes all cells in the involved field to high levels of oxidative stress resulting in formation of ROS, increasing DNA damage and ultimately leading to cell death [14]. Another mechanism of action of radiotherapy is to alter cellular homeostasis, modifying signal transduction pathways and disposition to apoptosis [14].

The nutritional status of HNSCC patients often reflects pre-diagnostic life style factors like smoking, drinking and low intake of fruits and vegetables, as well as changes in eating habits due to the tumour and adverse effects of radiotherapy. Although there have been advances in treatment, five-year survival rates in these patients has remained around 50-60% [10]. Some reports have shown that plasma levels of β-carotene, lycopene and vitamin E are lower in HNSCC [15-17] and oral leukoplakia patients [18,19] as compared to healthy controls. The effects of radiotherapy on antioxidants and oxidative stress biomarkers, and their association to survival are known to a much lesser extent. In a pilot study including 29 HNSCC patients, we observed that high levels of post-radiotherapy plasma total glutathione (GSH) [20] and carotenoids [21] were associated with increased survival in HNSCC patients.

We suggest that improved knowledge on the antioxidant and oxidative stress status, the impact of radiotherapy on this status, and its association with outcome could be useful for improving survival in these patients. Thus, in this study we have assessed an extensive profile of plasma low molecular weight antioxidants (endogenous and dietary) and oxidative stress markers before radiotherapy and monitored their respective response to radiotherapy in HNSCC patients. Furthermore, we have investigated how these parameters are related with survival.

## Methods

### Selection of patients and controls

Eighty-seven patients with histologically verified HNSCC were recruited from Division of Cancer Medicine and Radiotherapy, Norwegian Radium Hospital, Rikshospitalet University Hospital in the period from May 2003 to May 2006. Seventy-eight patients were found eligible (two patients did not receive complete radiotherapy and 7 patients had previous history of cancer) in this prospective study. Follow-up samples at the end of radiotherapy were obtained from 60 patients. Of these 60 patients, twenty-four received assisted nutrition during radiotherapy.

The patients received either post-operative radiotherapy or radiotherapy alone for a period of 5-7 weeks. All patients received external photon beam radiotherapy. The standard radiation technique used at the time of enrolment was computer tomography based conformal radiotherapy. Of the 78 patients included in the study, 50 patients received 70 Gy, 6 patients received 66 Gy, 1 patient received 64 Gy, 16 patients received 60 Gy (one died after 60 Gy) , 4 patients received 50 Gy and one patient died after 44 Gy. The patients receiving 70 Gy postoperatively had performed debulking surgery of the primary tumour and received 70 Gy to macroscopic lymph nodes in the neck and/or the primary tumour area. One patient received a split course accelerated hyperfractionated regimen with 1.5 Gy × 2 per day in 20 days (5 days per week). 76 patients received 2 Gy fractions 5 days weekly, but two of three patients with nasopharyngeal carcinomas received concomitant chemotherapy according to the Dahanca 14 protocol. The regimen used was cisplatin 40 mg/m^2 ^weekly and 6 fractions per week. One patient with nasopharyngeal carcinoma did not receive concomitant chemotherapy because of co-morbidity and high age. At the time of enrolment, concomitant chemotherapy was not the standard care at The Norwegian Radium Hospital.

Fasting plasma from 100 control individuals, matched with respect to age range, gender and smoking status with HNSCC patients were selected. These controls were selected from a cohort of 356 participants in a validation study conducted at the University of Oslo (Anette Karlsen, personal communication). Participants were recruited after response to an invitation letter that was sent to a random selection of citizens in the Norwegian capital city and surrounding area. The aims of the validation study were first to validate a new food frequency questionnaire, and secondly to investigate normal ranges of nutrition-related biomarkers measured in blood samples. The distribution of participants intended to reflect the general population and few exclusion criteria were defined. However, participants with self-reported diseases were excluded before the control group for this study was selected.

The basic characteristics of controls and patients are summarised in Table 1.

**Table 1:** Basic characteristics of patients and healthy controls

Parameters		Patients	Controls
Age (years)^a, b^		63 (34-85)	51 (34-80)
BMI^a^		24.8 (15.8-40.1)	25.8 (19.3-39.6)
Gender	Men	69	90
	Women	9	10
Tumour localization	Larynx	17	-
	Hypopharynx	9	-
	Oral cavity	21	-
	Oropharynx	28	-
	Nasopharynx	3	-
Stage	1	6	-
	2	20	-
	3	17	-
	4	35	-
Treatment	Surgery+radiotherapy	30	-
	Radiotherapy	48	-
Smoking status	Never smoker	28	34
	Former smoker	22	30
	Current smoker	28	36

^a^median (range)

^b^p < 0.001

The study was approved by The Regional Committee for Medical Research Ethics. All the patients and controls gave their written informed consent.

### Sample preparation

Blood samples taken before initiation of radiotherapy and in the last week of radiotherapy were collected in Stabilyte tubes and Vacutainer cell preparation tubes (CPT) containing sodium heparin as anticoagulant. Stabilyte tubes were centrifuged at 2800 g, 4°C for 10 minutes. The plasma obtained was prepared for reduced, oxidized and total GSH analysis as described in Sakhi et al. [22]. The CPT was centrifuged at 1750 g, 20°C for 20 minutes. For ascorbic acid analysis, plasma obtained was treated as described in Karlsen et al. [23]. For carotenoids, tocopherols, GGT, ferric reducing antioxidant power (FRAP), 8-isoprostagladin F_2α _(8-iso PGF_2α_) and total hydroperoxides (d-ROMs), plasma was portioned in 1 ml and snap frozen in liquid nitrogen and kept at -80°C until analysis. Blood samples from controls were taken in EDTA (for carotenoids and tocopherols) and sodium heparin tubes (for FRAP, d-ROMs and ascorbic acid).

### Analysis

The plasma levels of reduced and oxidized GSH were analysed as described by Sakhi et al. [24]. Total GSH and ascorbic acid (reduced and oxidized) were analysed as described by Bøhn et al. [20] and Karlsen et al. [23], respectively. Carotenoids and tocopherols were analysed as described in online supporting material by Karlsen et al. [25]. Two different modes of total antioxidant capacity were measured: FRAP and modified FRAP (FRAP without uric acid and proteins). The reason for measuring these two modes is that the role of uric acid, contributing 60% to FRAP measurements, as an endogenous antioxidant is inconclusive [26]. Thus, by measuring modified FRAP the effects on other plasma total antioxidants can be more clearly elucidated. FRAP was measured in untreated plasma by method described earlier [26]. For preparation of plasma extracts for modified FRAP, 10 μL uricase (0.1 units/10 μL) in Triz buffer (pH 8.5, 400 mmol/L) were added to 60 μL plasma. After incubation for 5 min at room temperature, 120 μL ethanol were added to precipitate proteins. Samples were placed at 4°C for 5 min before centrifugation at 13000 g at 4°C for 5 min. The uric acid- and protein free- supernatant were used for modified FRAP analysis.

Total hydroperoxides (d-ROMs) were measured in plasma by a d-ROM kit as described by the manufacture (Diacron International, Grosseto, Italy). Plasma γ-glutamyl transpeptidase (GGT) was analysed using Vitros GGT slides with a Vitros 950 Chemistry system at the Department of Clinical Chemistry, Norwegian Radium Hospital, Oslo University Hospital. 8-iso-PGF_2α _was analyzed by HPLC using MS-MS detector [27]. One ml plasma was hydrolyzed, acidified and concentrated using a C-18 solid phase extraction column. The eluate was dried and the residue was dissolved in 150 μL solution (water:methanol, 50:50, v/v). An aliquot of 50 μL sample was injected and separation was performed on two columns (SUPELCOSIL™ ABZ+Plus, 3 μm particle) connected in series. The mobile phases were (A) acetonitrile/formic acid (100:0.1, v/v) and (B) water/formic acid (100:0.1, v/v) at a flow rate of 0.35 mL/min. A gradient elution was used by increasing mobile phase A from 35% to 55% for 18 min, held for 2 min, and back to 35% mobile phase A in 2 min. MS-MS was operated in ESI negative mode. The precursor-to-product ion transitions in multiple reaction monitoring mode m/z 353.1-193.1 (for 8-iso PGF2α) and m/z 357.1-197.1 (for internal standard: 8-iso PGF2α-d4) were used for quantitation.

The redox potential of GSH in plasma was calculated using Nernst equation as described in Buettner et al. [28].

### Statistical analysis

The statistical program package SPSS (Statistical Package for Social Sciences, version 14.0) was employed. The biomarkers in pre-radiotherapy patients and controls were compared by independent samples t-tests. The groups were further compared, adjusted for age and body mass index (BMI) quartiles by Analysis of Covariance (ANCOVA). Paired t- tests were used to compare mean values before and after radiation therapy. Mean values are presented, and statistical significance was considered if p-value < 0.05.

We have used Cox' proportional hazards model to compare the survival distributions of groups with high and low levels of plasma biomarkers (cut off set to median value), and to obtain estimated hazard rate ratios.

To obtain adjusted hazard rate ratios, we tested the effect of a number of possible confounding factors: age, gender, smoking, treatment, stage, tumour localization and BMI. The confounding factors that changed the hazard rate ratios most were included in the final model. The number of confounding factors that could be included in the model depends on the sample size and we evaluated to have maximum 3 confounding factors in our study. Survival is shown by Kaplan-Meier plots, comparing patients with levels of biomarkers above and below the median.

For the correlation analyses, Pearson and Spearman's rho correlation coefficient were calculated.

For clinical outcome, progression-free and overall survivals have been used. Progression-free survival was calculated from start of radiotherapy until the date of first distant relapse, local recurrence, regrowth, regional lymph node metastasis, death from any cause or the last follow-up examination. Overall survival was calculated from the start of radiotherapy until death of any cause or last follow-up examination. Loco-regional control was calculated from start of radiotherapy until the date of local recurrence, regrowth, regional lymph node metastasis or the last follow-up examination.

All tests were done two-tailed and p-values < 0.05 considered statistically significant.

Due to different practical limitations, appropriate sample collection was not possible for every analysis in all patients and controls. Thus, the number of samples for pre-radiotherapy analysis for ascorbic acid, FRAP, modified FRAP, total GSH, total cysteine, GSH redox potential, d-ROMs, 8-iso PGF_2α _and ratio oxidized/total ascorbic acid (DHAA/TAA) were 73, 76, 76, 77, 77, 67, 76, 77 and 73 respectively. The number of samples for assessing the changes during radiotherapy for carotenoids, tocopherols, ascorbic acid, FRAP, modified FRAP, total GSH, total cysteine, GSH redox potential, d-ROMs, GGT, 8-iso PGF_2α _and ratio DHAA/TAA were 59, 59, 51, 57, 57, 57, 57, 50, 57, 59, 57 and 51, respectively. The numbers of control samples for analysis of tocopherols, FRAP, d-ROMs, total GSH and total cysteine were 97, 92, 99, 99 and 99, respectively. The remaining biomarkers had data for all patient and control samples.

## Results

### Plasma levels of antioxidants and oxidative stress biomarkers

#### HNSCC patients and controls

Mean plasma levels of antioxidants and oxidative stress biomarkers in healthy controls and HNSCC patients (both pre- and post- radiotherapy) are shown in Table 2 and 3. Pre-radiotherapy plasma levels of these biomarkers in HNSCC patients were compared to healthy controls. All carotenoids (lutein, zeaxanthin, β-cryptoxanthin, α-carotene, β-carotene, lycopene and total), α-tocopherol, ascorbic acid, FRAP, modified FRAP and total cysteine were significantly lower in HNSCC patients as compared to healthy controls. Total tocopherols also showed a trend and were lower in patients as compared to healthy controls (p = 0.07). For total carotenoids, total tocopherols, ascorbic acid, FRAP, modified FRAP and total cysteine the mean levels were 53%, 92% , 68%, 94%, 50% and 96% of the controls, respectively. Among oxidative stress parameters, d-ROMs were significantly higher (142%) in patients as compared to controls. Significant correlations were observed between stage of disease and pre-radiotherapy d-ROMs (r = 0.44, p < 0.001), and stage of disease and pre-radiotherapy total carotenoids (r = -0.31, p = 0.006) indicating that the patients with higher stage have high oxidative stress.

**Table 2:** Antioxidants in healthy controls and patients, both pre- and post radiotherapy

Antioxidants^a^		Controls	Patients	
			Pre-radiotherapy^b^	Post-radiotherapy^c^
Carotenoids (nmol/L)	Lutein	142.5	95.6^d^	51.0^d^
	Zeaxanthin	35.6	22.4^d^	12.2^d^
	β-cryptoxanthin	131.3	60.3^d^	27.5^d^
	α-carotene	107.6	61.3^d^	56.7^e^
	β-carotene	374.7	241.0^d^	229.5
	Lycopene	533.3	243.9^d^	124.6^d^
	Total carotenoids	1325.1	703.1^d^	474.5^d^
Tocopherols (μmol/L)	δ-tocopherol	0.030	0.080^d^	0.042^e^
	β-tocopherol	0.419	0.466	0.362^d^
	γ-tocopherol	1.70	1.72	1.06^d^
	α-tocopherol	28.3	25.7^e^	22.5^d^
	Total tocopherols	30.5	28.0	23.9^d^
Vitamin C (μmol/L)	Ascorbic acid	46.8	31.9^d^	23.3^d^
Total antioxidant capacity (μmol/L)	FRAP	1055	988^e^	979
	Modified FRAP	336	170^d^	166
Total GSH (μmol/L)		5.94	5.87	5.63
Total cysteine (μmol/L)		247.1	236.6^e^	235.3
GSH redox potential (mV)		n.a.	-148	-147

^a^Mean values

^b^Pre-radiotherapy levels are compared with healthy controls after adjusting for age and BMI

^c^Pre- and post- radiotherapy levels are compared

^d^p < 0.001, ^e^p < 0.05

n.a. = not available

**Table 3:** Oxidative stress biomarkers in healthy controls and patients, both pre- and post radiotherapy

Oxidative stress biomarkers^a^	Controls	Patients	
		Pre-radiotherapy^b^	Post-radiotherapy^c^
d-ROMS (CarrU)	155.9	219.6^d^	249.8^d^
GGT (U/L)	n.a.	38.9	59.4^e^
8-iso PGF_2α_ (pg/mL)	n.a.	28.1	30.9^e^
DHAA/TAA	0.085	0.089	0.116

^a^Mean values

^b^Pre-radiotherapy levels are compared with healthy controls after adjusting for age and BMI

^c^Pre- and post- radiotherapy levels are compared

^d^p < 0.001, ^e^p < 0.05

n.a. = not available

#### Changes during radiotherapy

Following radiotherapy, we observed a significant decline in plasma levels of dietary antioxidants including all carotenoids (except β-carotene), tocopherols and ascorbic acid, (Table 2). The mean decline for total carotenoids, total tocopherols and ascorbic acid were 33%, 15% and 27%, respectively. Twenty-four patients received assisted enteral nutrition fortified with β-carotene (130 μg/ml) during radiotherapy. A sub-group analysis excluding patients that received enteral nutrition also showed a decline for β-carotene (29%, p = 0.06). FRAP, modified FRAP and endogenous antioxidants (GSH redox potential, total GSH and cysteine) did not change significantly during treatment. The oxidative stress markers, d-ROMs, 8-iso PGF_2α _and GGT increased 14%, 10% and 53%, respectively during radiotherapy (Table 3).

### Clinical outcome

With a median follow-up time for survivors of 28 months (range 11-48 months), twenty-five patients are dead. Twenty-three patients died of HNSCC, one patient died of lung cancer and one patient died of unknown cause. Thirty-five patients have experienced progression: ten relapsed patients are alive, eighteen relapsed patients are dead, and seven patients are dead without previous relapse or regrowth of tumour. The projected three-year progression-free and overall survivals were 55% and 68%, respectively. Table 4 shows mean survival time estimates in months in all patients, only radiotherapy patients and post-operative radiotherapy patients for progression-free survival, overall survival and loco-regional control.

**Table 4:** Survival time estimates in months for progression-free survival, overall survival and loco-regional control.

Survival^a^	All patients n = 78	Only radiotherapy n = 48	Post-operative radiotherapy n = 30
Progression-free	28.1 (23.8-32.4)	23.8 (18.2-29.4)	31.5 (26.0-36.9)
Overall	34.7 (30.6-38.9)	29.1 (23.9-34.3)	42.8 (38.4-47.2)
Loco-regional control	32.8 (28.6-37.1)	29.2 (23.4-35.0)	34.5 (29.7-39.4)

The survival time is calculated in all patients, only radiotherapy patients and post-operative radiotherapy patients.

^a^Mean (95% confidence interval)

#### Pre-radiotherapy levels

**Figure 1:**
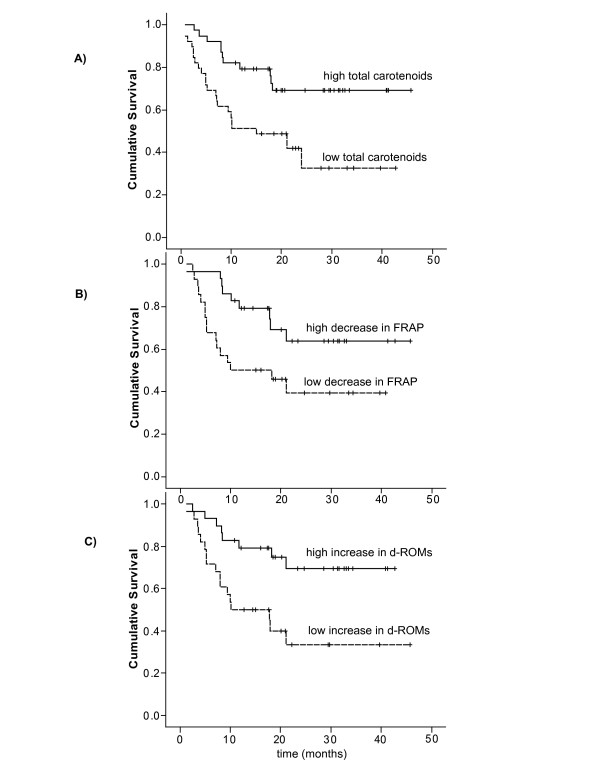
**Kaplan-Meier plot showing progression-free survival in patients**. A) Pre-radiotherapy plasma total carotenoids above median (high total carotenoids) versus plasma total carotenoids below median values (low total carotenoids), log rank p = 0.003; B) Relative decrease in plasma FRAP above median (high decrease in FRAP) versus relative decrease in plasma FRAP below median (low decrease in FRAP), log rank p = 0.024 and C) Relative increase in plasma d-ROMs above median (high increase in d-ROMs) versus relative increase in plasma d-ROMs below median (low increase in d-ROMs), log rank p = 0.006.

Above median values of several plasma carotenoids were significantly associated with improved progression-free survival. In univariate regression analysis of progression-free survival, high pre-radiotherapy plasma levels (above median) of total carotenoids and the single carotenoids (lutein, zeaxanthin, β-cryptoxanthin and β-carotene) were significantly associated with prolonged progression-free survival (Table 5). Plasma α-carotene did also show a close to significant association with prolonged progression-free survival (p = 0.06). In multivariate analyses adjusting for stage, treatment and smoking, these carotenoids remained significantly associated to survival (Table 5). The hazard rate ratio, representing the relative risk, was 0.5 for most of the carotenoids after adjusting for confounding factors showing that the patients with plasma carotenoid levels above median have 50% less risk of progression as compared to patients with plasma carotenoid levels below the median. Figure 1A presents Kaplan-Meier plot showing association of total carotenoids with progression-free survival. Similar results were observed for overall survival as shown in Table 5.

**Table 5:** Cox regression analysis showing association between survival and pre-radiotherapy plasma carotenoid levels.

Carotenoids	Crude			Adjusted^a^		
	Hazard rate ratio	95% CI	p-value	Hazard rate ratio	95% CI	p-value
Progression-free survival						

Lutein	0.40	0.20-0.80	0.01	0.47	0.22-0.98	0.04
zeaxanthin	0.49	0.25-0.97	0.04	0.55	0.27-1.09	0.09
β-cryptoxanthin	0.41	0.20-0.83	0.01	0.46	0.23-0.94	0.03
α-carotene	0.52	0.26-1.04	0.06	0.57	0.28-1.17	0.12
β-carotene	0.41	0.21-0.83	0.01	0.45	0.22-0.92	0.03
Lycopene	0.66	0.34-1.30	0.23	0.71	0.35-1.41	0.32
Total carotenoids	0.36	0.17-0.73	0.005	0.42	0.20-0.91	0.03

Overall survival						

Lutein	0.30	0.12-0.72	0.007	0.39	0.16-0.96	0.04
zeaxanthin	0.40	0.17-0.93	0.03	0.49	0.21-1.14	0.10
β-cryptoxanthin	0.25	0.10-0.63	0.003	0.27	0.11-0.69	0.006
α-carotene	0.32	0.14-0.78	0.01	0.36	0.15-0.86	0.02
β-carotene	0.20	0.07-0.52	0.001	0.23	0.08-0.62	0.004
Lycopene	0.63	0.28-1.40	0.26	0.63	0.27-1.45	0.28
Total carotenoids	0.26	0.10-0.65	0.004	0.38	0.15-0.98	0.05

The above median levels- are compared with below median levels- of plasma carotenoids in 78 patients for progression-free and overall survivals.

^a^The analysis are adjusted for stage, treatment and smoking status

No association with survival was observed for any other antioxidant- or oxidative stress biomarkers.

#### Changes during radiotherapy

Patients with a high relative decrease in the plasma levels of total antioxidant biomarker FRAP during radiotherapy have a prolonged progression-free survival as shown in figure 1B, that remained significant after adjusting for confounding factors (p = 0.05) (Table 6). No significant associations between progression-free survival and relative decrease in plasma levels of any other dietary or endogenous antioxidants during radiotherapy were observed.

**Table 6:** Cox regression analysis showing association between survival and relative changes in biomarkers.

Variables	Crude			Adjusted		
	Hazard rate ratio	95% CI	p-value	Hazard rate ratio	95% CI	p-value
Progression-free survival						

d-ROMs increase	0.33	0.14-0.76	0.01	0.31	0.13-0.76	0.01^a^
8-iso PGF_2α_ increase	0.48	0.22-1.08	0.08	0.51	0.22-1.17	0.11^b^
FRAP decrease	0.43	0.19-0.98	0.05	0.42	0.17-0.998	0.05^c^

Overall survival						

d-ROMs increase	0.26	0.08-0.80	0.02	0.31	0.10-1.00	0.05^a^
8-iso PGF_2α_ increase	0.54	0.21-1.42	0.21	0.58	0.21-1.59	0.29^b^
FRAP decrease	0.39	0.14-1.10	0.07	0.52	0.18-1.56	0.25^c^

The above median relative changes in plasma levels of oxidative stress biomarkers (d-ROMs and 8-iso PGF_2α_) and total antioxidant capacity FRAP are compared with below median relative changes in the plasma levels of the corresponding biomarkers in 57 patients for progression-free and overall survivals.

^a^The analysis are adjusted for stage, smoking status and tumour localization

^b^The analysis are adjusted for stage, smoking status and age

^c^The analysis are adjusted for stage, tumour localization and BMI

Patients with a high relative increase (above median) in the plasma levels of oxidative stress biomarker d-ROMs have a prolonged progression-free survival (Figure 1C). The association remained significant after adjusting for confounding factors (Table 6). The hazard rate ratio was as low as 0.3 showing that the patients with above median increase in d-ROMs during treatment period have 70% less risk of progression as compared to patients with under median increase in d-ROMs. Similar results were observed for overall survival as shown in Table 6. Among other oxidative stress biomarkers, a high relative increases in plasma levels of 8-iso PGF_2α _also showed a trend with prolonged progression-free survival (hazard rate ratio = 0.48, p = 0.08). After adjusting for confounding factors, the results were similar showing that there is a trend for 50% less risk of progression for patients with above median increase in plasma levels of 8-iso PGF_2α _during radiotherapy (Table 6).

In order to investigate a combined effect of high levels of total carotenoids and high increase in d-ROMs compared to low levels of both these factors (above median levels compared to below median levels), we utilized a two-factor Cox model. The interaction term turned out non-significant and was removed from the model. The risk of progression (hazard rate ratio: 0.21, 95% CI: 0.07-0.61) and overall death (hazard rate ratio: 0.13, 95% CI: 0.03-0.52), respectively, were 80% and 87% less for the patients with high level of total carotenoids and high increase in d-ROMs as compared to low levels of both these factors.

## Discussion

HNSCC patients have significantly lower levels of pre-radiotherapy plasma dietary antioxidants, FRAP and modified FRAP as compared to healthy controls; and dietary antioxidants further declined significantly during radiotherapy. The oxidative stress biomarker, d-ROMs, was significantly higher in patients as compared to controls and increased significantly during radiotherapy. Furthermore, pre-radiotherapy plasma carotenoids, relative increase in plasma levels of d-ROMs and relative decrease in plasma levels of FRAP during radiotherapy were positively associated with survival.

While low levels of pre-radiotherapy plasma β-carotene, lycopene and vitamin E have been suggested in some preliminary studies involving HNSCC patients [15-17], the present study is the first study where an extensive profile of antioxidants are measured in HNSCC patients and compared to healthy controls. The observed lower levels of plasma carotenoids in HNSCC patients are in accordance with the observation that low intake of carotenoid-rich fruits and vegetables increase the risk of cancers in the mouth, pharynx and larynx [10].

We also observed that these patients have high oxidative stress prior to radiotherapy as reflected by significantly higher plasma levels of d-ROMs as compared to healthy controls. To our knowledge, this is the first observation suggesting increased oxidative stress in newly diagnosed HNSCC patients before treatment.

All the dietary antioxidants measured decreased after radiotherapy. The decrease in plasma dietary antioxidants during radiotherapy is possibly a combination of reduced intake of antioxidant-rich foods (i.e., due to eating problems caused by adverse effects of radiotherapy, or decreased intake of fruits and vegetables) and increased utilization of antioxidants by free radicals produced from ionising radiations. Bäckström et al. [29] has shown that HNSCC patients experiencing dry mouth symptoms during radiotherapy have lower dietary intakes of β-carotene and vitamin E. In our cohort of patients there was a significant weight loss during the treatment period (median weight loss = 3.7%, p < 0.001). However, no significant correlation between weight loss and reduction in plasma antioxidant levels were observed (data not shown) suggesting that dietary intake in general does not account for this decline in plasma antioxidant levels. Thus, a specific decreased intake of antioxidant-rich foods and increased utilization of antioxidants during radiotherapy may be the plausible explanations for this decline. Increased utilization is in accordance with the observed increase in oxidative stress biomarkers, d-ROMs, 8-iso-PGF_2α _and GGT during radiotherapy.

We have investigated the role of a number of plasma antioxidants and oxidative stress biomarkers on survival in HNSCC patients. Our results demonstrated that among different dietary antioxidant biomarkers, pre-radiotherapy plasma lutein, β-cryptoxanthin, β-carotene and total carotenoids are strong prognostic factors for progression-free and overall survivals. The risk of progression was 60% less in patients having plasma carotenoids above median levels as compared to patients with under median levels. After correcting for various confounding factors (stage, treatment and smoking status), the relative risk remained close to 50%. Meyer et el. [30] also shows that high levels of plasma pre-radiotherapy β-carotene reduces rates of local tumour recurrence in head and neck cancer patients. This specific association of carotenoids with survival among dietary antioxidants may be due to difference in their chemical structure, compartmentalization and function as compared to other dietary antioxidants. Carotenoids are quenchers of singlet oxygen and are involved in restoring gap junctional communication (GJC) [31]. GJC is lost during carcinogenesis and may be important for malignant transformation. Carotenoids have shown to stimulate GJC in a differential and dose dependent manner although the underlying mechanisms are not fully understood [31].

We have previously shown that high levels of post-radiotherapy plasma carotenoids (lutein, α-carotene and β-carotene) were positively associated with survival in HNSCC patients [21]. Thus, plasma levels of several carotenoids, measured both before and after radiotherapy, are positively associated with survival in HNSCC patients.

Relative changes in plasma levels of d-ROMs and FRAP during radiotherapy showed an association with survival. The risk of progression developing distant relapse, local recurrence, regrowth or death was decreased by 70% and 60% for patients with an above median increase in d-ROMs and above median decrease in FRAP respectively, during treatment period. Relative changes in plasma levels of oxidative stress biomarker for lipid peroxidation, 8-iso PGF_2α _shows similar trend with survival after adjusting for confounding factors (p = 0.11). The risk of progression was decreased by 50% for patients with an above median increase in plasma levels of 8-iso PGF_2α_. Both d-ROMs and 8-iso PGF_2α _are biomarkers for oxidative stress, but measure total and specific oxidative damage in the cells, respectively. d-ROMs measure systemic oxidative damage caused by ROS to a number of organic substrates in a cell (e.g. lipids, amino acids, proteins, nucleotides and carbohydrates), but 8-iso PGF _2α _measures a more specific oxidative damage mainly to polyunsaturated fatty acids in the cells. The therapeutic effect of radiotherapy mainly comes from production of ROS in tumour cells. Our results, thus, indicate that patients with an increase in systemic oxidative stress during radiotherapy had better progression-free survival suggesting that increased oxidative stress may be beneficial during treatment period. We also observed that the risk of progression and overall death, respectively, were 80% and 87% less for the patients with high level of total carotenoids and high increase in d-ROMs as compared to low levels of both these factors (above median levels compared to below median levels). The strong combined adverse effect of low total carotenoid level and low increase in d-ROMs may identify a high-risk group of patients that could be candidates for an intervention trial.

Our data shows that high pre-radiotherapy plasma total carotenoid status is beneficial for HNSCC patients. Plasma total carotenoids are biomarkers of fruits and vegetable intake. Plasma carotenoids may also reflect intake of carotenoid supplement. However, β-carotene supplements which increase plasma β-carotene significantly, show no beneficial effects on mortality and development of second primary tumours in head and neck cancer patients [32,33], and even increase the risk of developing lung cancer [10]. Thus, increased intake of carotenoids through fruits and vegetables and not supplements in the period before radiotherapy may be beneficial for HNSCC patients.

Administration of carotenoid-rich fruits and vegetables may be more problematic during radiotherapy since oxidative stress mediates the therapeutic effects of radiotherapy and we observed that increased oxidative stress was beneficial during radiotherapy. To our knowledge, no study to date has studied the effect of increased carotenoid-rich fruits and vegetables during radiotherapy on the survival in this group of patients. Data from intervention studies with supplements (both α-tocopherol and β-carotene) in head and neck cancer patients during and after radiotherapy showed the supplement group had less severe side-effects of radiotherapy, but showed trends for increased rates of recurrence [34] that were significant in smokers [35]. Reasons for increased rates of recurrence could be that the use of supplements and smoking reduce radiation treatment efficacy.

## Conclusion

Our data shows that plasma levels of pre-radiotherapy carotenoids in HNSCC patients are lower than healthy controls, decrease during radiotherapy and are positively associated with progression-free and overall survivals. High oxidative stress during treatment period is also beneficial for survival. The possible association between intake of carotenoid-rich fruits and vegetables and survival of HNSCC patients before, during and after radiotherapy should be investigated in future studies.

## Abbreviations

HNSCC: Head and neck squamous cell carcinoma; FRAP: ferric reducing antioxidant power; d-ROMs: total hydroperoxides; ROS: reactive oxygen species; RNS: reactive nitrogen species; GSTs: glutathione S-transferases; HPV: human papillomavirus; GSH: glutathione; CPT: cell preparation tubes; 8-iso PGF_2α_: 8-isoprostagladin F_2α_; GGT: gamma-glutamyl transpeptidase; BMI: body mass index; ANCOVA: Analysis of Covariance; DHAA/TAA: oxidized/total ascorbic acid.

## Competing interests

RB has an interest in the companies Cgene AS, Bioindex AS and Vitas AS.

Cgene and Bioindex were established by Birkeland Innovation, the Technology transfer office at the University of Oslo while Vitas was established by the Oslo Innovation Center. NEB works part time in the company Vitas AS. AKS worked part time for 2 months (January and February 2008) in the company Vitas AS.

The other authors declare that they have no competing interests.

## Authors' contributions

AKS and KMR were responsible for sample collection, acquisition of patient data, statistical analysis, drafting and revising of the manuscript. AKS also executed the biomarker analysis in patient samples. MT was responsible for the statistical analysis and revising of the manuscript. NEB did the isoprostane analysis in patient samples. AK was responsible for the acquisition and analysis of control samples and revising of the manuscript. SS and RB planned the study and revised the manuscript. All authors read and approved the final manuscript.

## Pre-publication history

The pre-publication history for this paper can be accessed here:

http://www.biomedcentral.com/1471-2407/9/458/prepub

## References

[B1] Halliwell B Gutteridge JM Free Radicals in Biology and Medicine 2007 Fourth New York: Oxford University Press Inc

[B2] Lasheras C Huerta JM Gonzalez S Brana AF Patterson AM Fernandez S Independent and interactive association of blood antioxidants and oxidative damage in elderly people Free Radic Res 2002 36 875 882 10.1080/1071576021000005311 12420746

[B3] Yeum KJ Russell RM Krinsky NI Aldini G Biomarkers of antioxidant capacity in the hydrophilic and lipophilic compartments of human plasma Arch Biochem Biophys 2004 430 97 103 10.1016/j.abb.2004.03.006 15325916

[B4] Serafini M Villano D Spera G Pellegrini N Redox molecules and cancer prevention: the importance of understanding the role of the antioxidant network Nutr Cancer 2006 56 232 240 10.1207/s15327914nc5602_15 17474870

[B5] Blomhoff R Dietary antioxidants and cardiovascular disease Curr Opin Lipidol 2005 16 47 54 10.1097/00041433-200502000-00009 15650563

[B6] Valko M Rhodes CJ Moncol J Izakovic M Mazur M Free radicals, metals and antioxidants in oxidative stress-induced cancer Chem Biol Interact 2006 160 1 40 10.1016/j.cbi.2005.12.009 16430879

[B7] Halliwell B Oxidative stress and cancer: have we moved forward? Biochem J 2007 401 1 11 10.1042/BJ20061131 17150040

[B8] Toyokuni S Oxidative stress and cancer: the role of redox regulation Biotherapy 1998 11 147 154 10.1023/A:1007934229968 9677046

[B9] Sturgis EM Wei Q Spitz MR Descriptive epidemiology and risk factors for head and neck cancer Semin Oncol 2004 31 726 733 10.1053/j.seminoncol.2004.09.013 15599850

[B10] World Cancer Research Fund/American Institute for Cancer Research Food, Nutrition, Physical Activity, and the Prevention of Cancer: a Global Perspective 2007 Washington DC: AICR

[B11] Ye Z Song H Guo Y Glutathione S-transferase M1, T1 status and the risk of head and neck cancer: a meta-analysis J Med Genet 2004 41 360 365 10.1136/jmg.2003.016246 15121774 PMC1735789

[B12] Mork J Lie AK Glattre E Hallmans G Jellum E Koskela P Human papillomavirus infection as a risk factor for squamous-cell carcinoma of the head and neck N Engl J Med 2001 344 1125 1131 10.1056/NEJM200104123441503 11297703

[B13] D'Souza G Kreimer AR Viscidi R Pawlita M Fakhry C Koch WM Case-control study of human papillomavirus and oropharyngeal cancer N Engl J Med 2007 356 1944 1956 10.1056/NEJMoa065497 17494927

[B14] Borek C Antioxidants and radiation therapy J Nutr 2004 134 3207S 3209S 15514309 10.1093/jn/134.11.3207S

[B15] Kune GA Kune S Field B Watson LF Cleland H Merenstein D Oral and pharyngeal cancer, diet, smoking, alcohol, and serum vitamin A and beta-carotene levels: a case-control study in men Nutr Cancer 1993 20 61 70 10.1080/01635589309514271 8415131

[B16] Cartmel B Bowen D Ross D Johnson E Mayne ST A randomized trial of an intervention to increase fruit and vegetable intake in curatively treated patients with early-stage head and neck cancer Cancer Epidemiol Biomarkers Prev 2005 14 2848 2854 10.1158/1055-9965.EPI-05-0191 16364999

[B17] Manoharan S Kolanjiappan K Suresh K Panjamurthy K Lipid peroxidation & antioxidants status in patients with oral squamous cell carcinoma Indian J Med Res 2005 122 529 534 16518005

[B18] Nagao T Ikeda N Warnakulasuriya S Fukano H Yuasa H Yano M Serum antioxidant micronutrients and the risk of oral leukoplakia among Japanese Oral Oncol 2000 36 466 470 10.1016/S1368-8375(00)00037-3 10964055

[B19] Ramaswamy G Rao VR Kumaraswamy SV Anantha N Serum vitamins' status in oral leucoplakias--a preliminary study Eur J Cancer B Oral Oncol 1996 32B 120 122 10.1016/0964-1955(95)00076-3 8736174

[B20] Bøhn SK Smeland S Sakhi AK Thoresen M Russnes KM Tausjø J Post-radiotherapy plasma total glutathione is associated to outcome in patients with head and neck squamous cell carcinoma Cancer Lett 2006 238 240 247 10.1016/j.canlet.2005.07.027 16157445

[B21] Sakhi AK Bøhn SK Smeland S Thoresen M Smedshaug GB Tausjø J Post-radiotherapy plasma lutein, alpha-carotene and beta-carotene are positively associated with survival in patients with head and neck squamous cell carcinoma Nutrition and Cancer: An International Journal 2009 in press 10.1080/01635580903441188 20358469

[B22] Sakhi AK Russnes KM Smeland S Blomhoff R Gundersen TE Simultaneous quantification of reduced and oxidized glutathione in plasma using a two-dimensional chromatographic system with parallel porous graphitized carbon columns coupled with fluorescence and coulometric electrochemical detection J Chromatogr A 2006 1104 179 189 10.1016/j.chroma.2005.11.129 16376913

[B23] Karlsen A Blomhoff R Gundersen TE High-throughput analysis of vitamin C in human plasma with the use of HPLC with monolithic column and UV-detection J Chromatogr B Analyt Technol Biomed Life Sci 2005 824 132 138 10.1016/j.jchromb.2005.07.008 16046288

[B24] Sakhi AK Blomhoff R Gundersen TE Simultaneous and trace determination of reduced and oxidized glutathione in minute plasma samples using dual mode fluorescence detection and column switching high performance liquid chromatography J Chromatogr A 2007 1142 178 184 10.1016/j.chroma.2006.12.051 17208244

[B25] Karlsen A Retterstol L Laake P Paur I Kjolsrud-Bohn S Sandvik L Anthocyanins inhibit nuclear factor-kappaB activation in monocytes and reduce plasma concentrations of pro-inflammatory mediators in healthy adults J Nutr 2007 137 1951 1954 17634269 10.1093/jn/137.8.1951

[B26] Benzie IF Strain JJ The ferric reducing ability of plasma (FRAP) as a measure of "antioxidant power": the FRAP assay Anal Biochem 1996 239 70 76 10.1006/abio.1996.0292 8660627

[B27] Bastani NE Gundersen TE Blomhoff R Determination of 8-epi PGF(2alpha) concentrations as a biomarker of oxidative stress using triple-stage liquid chromatography/tandem mass spectrometry Rapid Commun Mass Spectrom 2009 23 2885 2890 10.1002/rcm.4197 19670343

[B28] Schafer FQ Buettner GR Redox environment of the cell as viewed through the redox state of the glutathione disulfide/glutathione couple Free Radic Biol Med 2001 30 1191 1212 10.1016/S0891-5849(01)00480-4 11368918

[B29] Backstrom I Funegard U Andersson I Franzen L Johansson I Dietary intake in head and neck irradiated patients with permanent dry mouth symptoms Eur J Cancer B Oral Oncol 1995 31B 253 257 10.1016/0964-1955(95)00023-B 7492922

[B30] Meyer F Bairati I Jobin E Gelinas M Fortin A Nabid A Acute adverse effects of radiation therapy and local recurrence in relation to dietary and plasma beta carotene and alpha tocopherol in head and neck cancer patients Nutr Cancer 2007 59 29 35 17927499 10.1080/01635580701397590

[B31] Tapiero H Townsend DM Tew KD The role of carotenoids in the prevention of human pathologies Biomed Pharmacother 2004 58 100 110 10.1016/j.biopha.2003.12.006 14992791 PMC6361147

[B32] Mayne ST Cartmel B Baum M Shor-Posner G Fallon BG Briskin K Randomized trial of supplemental beta-carotene to prevent second head and neck cancer Cancer Res 2001 61 1457 1463 11245451

[B33] Toma S Bonelli L Sartoris A Mira E Antonelli A Beatrice F beta-carotene supplementation in patients radically treated for stage I-II head and neck cancer: results of a randomized trial Oncol Rep 2003 10 1895 1901 14534715

[B34] Bairati I Meyer F Gelinas M Fortin A Nabid A Brochet F Randomized trial of antioxidant vitamins to prevent acute adverse effects of radiation therapy in head and neck cancer patients J Clin Oncol 2005 23 5805 5813 10.1200/JCO.2005.05.514 16027437

[B35] Meyer F Bairati I Fortin A Gelinas M Nabid A Brochet F Interaction between antioxidant vitamin supplementation and cigarette smoking during radiation therapy in relation to long-term effects on recurrence and mortality: a randomized trial among head and neck cancer patients Int J Cancer 2008 122 1679 1683 10.1002/ijc.23200 18059031

